# Potential Fluid Biomarkers and a Prediction Model for Better Recognition Between Multiple System Atrophy-Cerebellar Type and Spinocerebellar Ataxia

**DOI:** 10.3389/fnagi.2021.644699

**Published:** 2021-04-20

**Authors:** Shuo Guo, Bi Zhao, Yunfei An, Yu Zhang, Zirui Meng, Yanbing Zhou, Mingxue Zheng, Dan Yang, Minjin Wang, Binwu Ying

**Affiliations:** ^1^Department of Laboratory Medicine, West China Hospital of Sichuan University, Chengdu, China; ^2^Department of Neurology, West China Hospital of Sichuan University, Chengdu, China; ^3^Department of Radiology, West China Hospital of Sichuan University, Chengdu, China

**Keywords:** multiple system atrophy, LASSO, prediction model, spinocerebellar ataxia, liquid biomarkers

## Abstract

**Objective:**

This study screened potential fluid biomarkers and developed a prediction model based on the easily obtained information at initial inspection to identify ataxia patients more likely to have multiple system atrophy-cerebellar type (MSA-C).

**Methods:**

We established a retrospective cohort with 125 ataxia patients from southwest China between April 2018 and June 2020. Demographic and laboratory variables obtained at the time of hospital admission were screened using Least Absolute Shrinkage and Selection Operator (LASSO) regression and logistic regression to construct a diagnosis score. The receiver operating characteristic (ROC) and decision curve analyses were performed to assess the accuracy and net benefit of the model. Also, independent validation using 25 additional ataxia patients was carried out to verify the model efficiency. Then the model was translated into a visual and operable web application using the R studio and Shiny package.

**Results:**

From 47 indicators, five variables were selected and integrated into the prediction model, including the age of onset (AO), direct bilirubin (DBIL), aspartate aminotransferase (AST), eGFR, and synuclein-alpha. The prediction model exhibited an area under the curve (AUC) of 0.929 for the training cohort and an AUC of 0.917 for the testing cohort. The decision curve analysis (DCA) plot displayed a good net benefit for this model, and external validation confirmed its reliability. The model also was translated into a web application that is freely available to the public.

**Conclusion:**

The prediction model that was developed based on laboratory and demographic variables obtained from ataxia patients at admission to the hospital might help improve the ability to differentiate MSA-C from spinocerebellar ataxia clinically.

## Introduction

Multiple system atrophy (MSA) is a sporadic and continuously progressive neurodegenerative disorder (Gilman et al., 2008). MSA includes two primary subtypes, predominant parkinsonism (MSA-P) and cerebellar ataxia (MSA-C), of which MSA-C is the most common subtype in the East-Asian population (Watanabe et al., 2002; Gilman et al., 2005; Yabe et al., 2006). Presently, there is no effective treatment for MSA-C, but clinical intervention in the early stages of the disease might improve patients’ quality of life and prolong their survival (Klockgether et al., 1998; Wenning et al., 2013; Jacobi et al., 2015; Fanciulli et al., 2019). Therefore, early diagnosis of MSA-C is the central focus of current research.

No specific and objective biomarkers are known for MSA-C. Disease history, clinical manifestations, neurological examinations, and some neuroimaging features are currently common methodologies used to diagnose MSA-C. However, due to individual patient differences and the disease stage, it is typically challenging to diagnose MSA-C accurately based on these conventional characteristics, and it is easy to confuse MSA-C with other ataxia diseases, specifically hereditary spinocerebellar ataxia (SCA) (Palma et al., 2018). Therefore, objective biomarkers properly useful for distinguishing between these two diseases would be of great help when initial clinical features are similar. Currently, numerous studies have focused on identifying candidate disease biomarkers for MSA-C from cerebrospinal fluid (CSF) and peripheral blood (Jellinger, 2017). CSF is an ideal biological sample because it is more likely to reflect specific neurophysiological changes, but it must be obtained through invasive surgery (lumbar puncture). On the other hand, peripheral blood is safer and easier to obtain. The various biomarkers in the blood including proteins, lipids, and many other metabolites could serve as potential diagnostic and prognostic markers for the disease.

The liquid biomarkers selected in our study were mainly divided into two groups. One group is related metabolic indicators which are actually clinical basic indicators routinely tested for diagnostic use. Previous studies have shown that abnormal metabolites change may exist in neurodegenerative diseases including Alzheimer’s disease (AD), Parkinson’s disease (PD), as well as MSA (Zhou et al., 2016; Nam et al., 2018; Takae et al., 2018; Nho et al., 2019). Notably, several studies have shown that the levels of metabolic related markers including uric acid (URIC) and homocysteine are aberrant in MSA patients (Lee et al., 2011; Chen et al., 2015; Zhou et al., 2016). Therefore, the screening of those markers reflecting the metabolic status of patients which are also widely available in clinical laboratories may provide potential clues for diagnosis and pathogenesis study of MSA. The other group includes proteins that are associated with inflammation, neurodegeneration, regeneration, and so on. Previous studies have indicated that the glial inflammation may play a role in MSA disease progression (Yokoyama et al., 2007). A study showed CSF cytokine/chemokine/growth factor profiles in MSA-C and SCA in which pro-inflammatory cytokines like IL-6, GM-CSF, and MCP-1 displayed specific correlation with the disease stage in MSA-C (Yamasaki et al., 2017). Besides, several proteins including calbindin D, amyloid precursor protein (APP), S100B, and synuclein-alpha (α-synuclein) have been ascertained in neurodegenerative diseases such as AD, Huntington’s disease (HD), multiple sclerosis, and MSA (Steiner et al., 2011; Stefanits et al., 2014; van Waalwijk van Doorn et al., 2016; Mavroudis et al., 2020). Meanwhile, the investigation of other proteins such as carbonic anhydrase, CD117/c-kit, proganulin, and kallikreins which may play roles in neural circuit development and maintenance, stress response, innate immunity, and aging as well as brain innate immunity may open a new avenue for the study of MSA (Greco et al., 2012; Dukic et al., 2016; Chitramuthu et al., 2017; Gennarini et al., 2017; Hsieh et al., 2019).

Despite the continuous exploration of specific biomarkers, recent efforts have been made on establishing clinical prediction models integrating demographic characteristics, clinical variables, and laboratory indicators for improving the diagnosis or predicting survival prognosis of neurological diseases with an output of quantitative risk estimate using limited number of relatively objective predictors. Therefore, we screened potential fluid biomarkers of MSA-C and combined mainly demographics characteristics to establish a clinical prediction model to improve the early identification and diagnosis of MSA-C.

## Materials and Methods

### Participants

Seventy-nine MSA-C patients and 46 hereditary ataxia patients were enrolled in the Department of Neurology, West China Hospital, Sichuan University, between April 2018 and June 2020. The MSA-C patients were assessed and defined based on the second consensus statement on the diagnosis of MSA, which is universally adopted (Gilman et al., 2008). Briefly, the MSA-C patients exhibited specific features: (1) sporadic, progressive, adult-onset disease signs (age > 30 years) with predominant cerebellar syndromes, including gait ataxia, dysarthria, limb ataxia, or cerebellar oculomotor dysfunction; (2) autonomic failure involving urinary incontinence, erectile dysfunction and orthostatic hypotension, or parkinsonism with a poor levodopa response; and (3) no common genetic diagnosis of hereditary ataxia. The patients diagnosed with hereditary ataxia were assessed based on the diagnostic criteria associated with SCA (Muzaimi et al., 2004; Klockgether et al., 2019). The diagnostic guidelines for hereditary SCA included (1) onset of symptoms that occurred in patients older than 18 and presented predominantly progressive cerebellar ataxia with a disease duration longer than 1 year; and (2) cases with a family history of the presence of a similar disorder, and after passing molecular genetic testing, it was determined that the patients carried SCA-related mutant genes. We have screened the gene for SCA1, SCA2, SCA3, SCA6, SCA7, SCA8, SCA10, SCA12, SCA17, and DRPLA. The results turned out that there were only SCA1, SCA2, SCA3, and SCA6 patients in our study.

**FIGURE 1:**
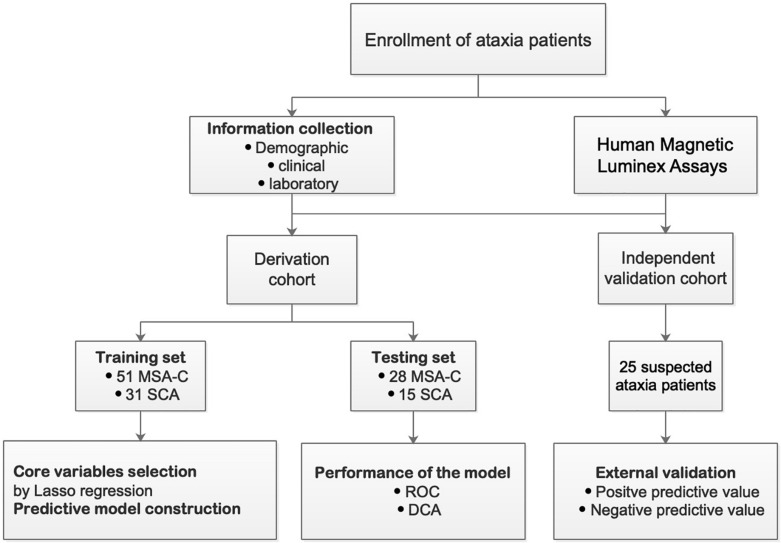
Flowchart of the study design.

Individuals were not included in the study if they exhibited secondary ataxia caused by cerebrovascular disease, tumors, alcoholism, vitamin B_1_ or B_12_ deficiency, folate deficiency, drug use, neurosyphilis, multiple sclerosis, paraneoplastic cerebellar degeneration, immune-mediated cerebellitis, or hypothyroidism. From August 2019 to October 2020, we included an additional 25 patients with undiagnosed ataxia in an independent verification cohort for evaluation and analysis. The schematic diagram for the research design is shown in Figure 1.

### Information on the Collection and Detection of the Fluid Biomarkers

Information was collected for each patient concerning their demographic and clinical characteristics as well as laboratory examination results when they were first admitted and before any treatment had occurred. The laboratory examination namely as related metabolic or biochemical indicators included total bilirubin (TBIL), direct bilirubin (DBIL), indirect bilirubin (IBIL), alanine aminotransferase (ALT), aspartate aminotransferase (AST), total protein (TP), albumin (ALB), globulin (GLB), urea (UREA), creatinine (CREA), cystatin C (CysC), URIC, triglyceride (TG), cholesterol (CHOL), high-density lipoprotein cholesterol (HDLC), low-density lipoprotein cholesterol (LDLC), alkaline phosphatase (ALP), glutamyl transpeptidase (GGT), estimated glomerular filtration rate (eGFR), sodium (NA), potassium (K), lactate dehydrogenase (LDH), hydroxybutyrate dehydrogenase (HBDH), creatine kinase (CK), and glucose (GLU). They are actually clinical basic indicators routinely tested for diagnostic use. These analytes were tested by qualified laboratory personnel following standard operating procedures established by the Department of Laboratory Medicine in West China Hospital of Sichuan University (WCH-LM-CHE-SOP-T1). Also, they were measured using Roche Cobas 702 automatic biochemical analyzer (Roche, Mannheim, Germany) with the corresponding reagents, calibrators, and quality control materials. The specific method for each analyte is listed in Supplementary Table 1.

Additional testing for 20 proteins included C-C motif ligand (CCL)2/macrophage chemoattractant protein-1 (MCP-1), CCL11, CD117/c-kit, α-synuclein, contactin-1, interleukin-1 receptor antagonist (IL-1ra), IL-1β, IL-6, IL-15, IL-7, GM-CSF, carbonic anhydrase, S100B, APP, calbindin D, proganulin, kallikrein 3, kallikrein 5, kallikrein 6/neurosin, and urokinase. These proteins were detected using Human Magnetic Luminex Screening Assay (LXSAHM; R&D Systems, Minneapolis, MN, United States) on Bio-Plex 200 detection platform (Bio-Rad, California, United States) according to the manufacturer’s instructions. The serum samples for Luminex assays were the residuals of blood samples obtained from patients for routine clinical experiments at first admission. They were centrifuged for 15 min at 1,000 × *g* then were stored at −80°C until used. On the day the samples were assessed, previously frozen serum samples were centrifuged at 16,000 × *g* for 4 min immediately and 50 μl of serum samples were handled in twofold dilutions with Calibrator Diluent RD6-52 provided in the kit. The sample concentration was calculated based on the standard curve determined for each analyte, which was derived from the serial dilution concentration of the standard. No sample exceeded the upper detection limit or fell below the lower detection limit. The standards were tested in duplicate. As for the standard curve, the coefficient of variation (CV) was calculated and did not exceed 20% and the recovery rate was between 80 and 120%. The detailed principles and protocols are introduced in Supplementary Sheet 1.

### Core Variable Selection and Identification of the Established Model

The Least Absolute Shrinkage and Selection Operator (LASSO) regression analysis was performed to select core variables that could decrease the regression coefficient for each variable within a specific range and eliminate the feature with a coefficient of 0, independent of statistical significance (Tibshirani, 1997). Forty-seven possible indicators including AO, gender, 25 related metabolic markers, and 20 proteins were included in LASSO analysis at first. This protocol identified variables that were more representative for disease outcomes that allowed the identification of an optimally refined generalized linear model without overfitting, which was better suited for the variable analysis of studies with small sample numbers (Corey et al., 2018). The remaining core variables were integrated to establish a model using logistic regression. Shiny R Package was used to build interactive web applications. The steps described previously were accomplished using R, version 3.5.0, for Mac.

### Statistical Analysis

The distributions of variables were assessed using Kolmogorov–Smirnov tests and quantile–quantile plots. Continuous variables with normal distribution were presented as mean ± SDs. Continuous variables not following the normal distribution and categorical variables were presented as medians (upper and lower quartiles) and in terms of frequency, respectively. The χ^2^ test for categorical variables and Student’s *t*-test or Mann–Whitney *U* test for continuous variables were applied to compare the two groups. The diagnostic performance of the equation was displayed using receiver operating characteristic (ROC) analysis and quantified using the area under the curve (AUC). Decision curve analysis (DCA) was used to measure the net clinical benefits. All statistical analyses were carried out using SPSS, version 25.0, and R, version 3.5.0, for Mac. All statistical tests were two-tailed, and *P* <0.05 indicated statistical significance.

### Standard Protocol Approvals, Registrations, and Patient Consent

The protocols used in this study were approved by the West China Hospital, Sichuan University Medical Ethics Committee. Written informed consent was obtained from all participants.

## Results

### Demographic and Clinical Information

One hundred twenty-five patients were included in a derivation cohort, among which 82 patients (31 SCA vs. 51 MSA-C) were enrolled randomly in a training cohort, and 43 patients (15 SCA vs. 28 MSA-C) were enrolled randomly in a testing cohort. The frequency of MSA-C in the training cohort (62.20%) was not significantly different from the testing cohort (65.12%). Also, medical information from an additional 25 ataxia-like patients was collected using the same criteria for external independent validation. The demographic and clinical characteristics of participants in the derivation cohort are shown in Table 1. The median age of onset (AO) for MSA-C and SCA was significantly different. The information of different subtypes of SCA patients are displayed in Supplementary Table 2.

**TABLE 1:** Demographic and clinical characteristics of the patients enrolled.

	Training cohort			Testing cohort		
	MSA-C	SCA	*P*	MSA-C	SCA	*P*
Age of onset	59(53–65)	43(36–54)	**<0.001**	56(53–64)	49(37–52)	**0.004**
Gender	26/25	14/17	0.609	15/13	9/6	0.813
Family history	0/45	26/30	**<0.001**	0/25	12/15	**<0.001**
Autonomic dysfunction	49	15	**<0.001**	25	8	**0.023**
Atrophy on MRI	47	26	0.424	26	11	0.161

*Gender was presented as *male/female*; Family history was shown as *cases with positive family history/total cases with family history inquire*; Atrophy on MRI refers to atrophy on MRI of putamen, middle cerebellar peduncle, pons, or cerebellum. The bold values represent the P value less than 0.05.*

Among the fluid markers assessed in the training set, we observed only IL-7 as a neuroinflammation-related cytokine that was significantly differentially expressed between MSA-C patients and SCA patients, with higher levels in SCA patients (Table 2 and Supplementary Figure 1). Similarly, four metabolites exhibited different levels between the two groups, including relatively increased AST, GLU, and CysC, while the level of eGFR was lower in MSA-C patients. However, different trends of expressed markers were observed in the testing set where additional metabolomic changes existed (Table 2).

**TABLE 2:** The fluid biomarkers levels of the patients enrolled.

	Training cohort			Testing cohort		
	MSA-C	SCA	*P*	MSA-C	SCA	*P*
TBIL (μmol/L)	11.5(8.6–14.2)	11.2(9.4–15.8)	0.334	11.5(8.1–14.7)	13.3(9.2–15.6)	0.199
DBIL (μmol/L)	3.3(2.8–4.2)	3.6(2.9–5.6)	0.144	3.1(2.7–3.5)	3.3(3.0–3.8)	0.189
IBIL (μmol/L)	7.4(5.5–9.9)	7.9(6–10.3)	0.503	7.6(5.2–9.6)	9.3(6.0–11.0)	0.186
ALT (IU/L)	18(14–24)	14(11–23)	0.127	19(16–25)	25.5(17–39.5)	0.198
AST (IU/L)	21(18–28)	19(15–23)	**0.027**	26(21.5–32)	20(18–25)	**0.002**
TP (g/L)	69.19 ± 5.57	71.36 ± 5.99	0.101	67.38 ± 6.1	73.34 ± 5.77	**0.001**
ALB (g/L)	43.91 ± 3.92	44.46 ± 4.00	0.537	41.98 ± 3.69	44.99 ± 3.56	**0.005**
GLB (g/L)	25.28 ± 4.48	26.89 ± 4.26	0.112	25.40 ± 3.74	28.35 ± 4.04	**0.009**
GLU (mmol/L)	5.07(4.62–5.75)	4.77(4.33–5.15)	**0.041**	5.07(4.62–5.71)	4.71(4.35–5.62)	0.331
UREA (mmol/L)	5.17 ± 1.57	4.67 ± 1.31	0.147	5.40 ± 1.72	5.02 ± 0.77	0.36
CREA (μmol/L)	63(55–75)	59(50–67)	0.085	62(55–83)	79(53–81)	0.869
CysC (mg/L)	0.91 ± 0.11	0.82 ± 0.15	**0.002**	0.94 ± 0.18	0.92 ± 0.14	0.696
URIC (μmol/L)	282(241–369)	275(225–327)	0.253	314(256–384)	293(229–370)	0.474
TG (mmol/L)	1.44 ± 0.96	1.17 ± 0.52	0.158	1.48 ± 0.76	1.32 ± 0.74	0.446
CHOL (mmol/L)	4.55 ± 0.78	4.57 ± 0.76	0.887	4.37 ± 0.92	4.60 ± 0.96	0.39
HDLC (mmol/L)	1.50 ± 0.42	1.50 ± 0.44	0.928	1.20 ± 0.36	1.35 ± 0.37	0.174
LDLC (mmol/L)	2.58 ± 0.68	2.72 ± 0.70	0.374	2.58 ± 0.73	2.68 ± 0.76	0.557
ALP (IU/L)	69(61–89)	69(59–83)	0.473	78(68–92)	85(67–96)	0.373
GGT (IU/L)	21(13–35)	15(13–22)	0.121	20(16–32)	22(15–34)	0.912
eGFR (mL/min)	95.50 ± 11.73	111.15 ± 12.46	**< 0.001**	96.04 ± 14.03	101.27 ± 18.58	0.284
NA (mmol/L)	142.66 ± 1.82	142.07 ± 2.16	0.189	143.68 ± 1.70	142.76 ± 2.44	0.135
K (mmol/L)	4.10(3.87–4.4)	4.09(3.99–4.27)	0.996	3.97(3.75–4.12)	3.93(3.67–4.11)	0.588
LDH (IU/L)	177(152–198)	178(159–197)	0.977	161(144–182)	191(162–211)	**0.002**
HBDH (IU/L)	142(118–156)	144(121–155)	0.935	126(111–139)	153(130–169)	**0.001**
CK (IU/L)	75(59–101)	85(66–126)	0.16	80(53–109)	107(68–152)	**0.033**
Carbonic Anhydrase (pg/mL)	28.59 ± 16.33	37.27 ± 22.33	0.229	24.41 ± 19.58	31.51 ± 18.02	0.345
Proganulin (pg/mL)	41965.39 ± 16410.80	42880.48 ± 18336.43	0.885	37814.59 ± 19829.00	45736.21 ± 18312.81	0.282
Urokinase (pg/mL)	746.88 ± 448.37	793.91 ± 342.10	0.744	736.29(328.83–959.34)	766.57(257.69–920.59)	0.91
APP (pg/mL)	4863.79 ± 2041.30	6014.14 ± 3215.31	0.125	8463.76 ± 3176.12	10612.37 ± 3202.44	0.099
S100B (pg/mL)	477.98 ± 95.71	547.85 ± 145.75	0.151	350.31 ± 222.26	522.11 ± 327.68	0.117
Calbindin D (pg/mL)	46.91 ± 18.48	52.20 ± 24.47	0.528	61.97 ± 26.31	65.95 ± 23.07	0.673
Contactin-1 (pg/mL)	95.41(71.59–119.41)	109.49(76.70–137.85)	0.643	102(78–133)	148.90(99.61–195.40)	**0.042**
GM-CSF (pg/mL)	9.95(8.33–12.85)	14.71(8.87–27.42)	0.077	11.75(9.82–17.99)	15.71(12.49–20.04)	0.16
CCL11 (pg/mL)	139.04 ± 81.04	123.34 ± 69.19	0.592	177.52 ± 110.48	137.34 ± 78.29	0.31
CCL2/MCP-1 (pg/mL)	1886.92(1299.49–2502.96)	1892.47(1255.21–2380.58)	0.981	1772.44(1285.95–2700.49)	2388.32(1388.61–2643.39)	0.285
CD117/c kit (pg/mL)	2871.91(1707.97–4055.16)	2806.98(1728.85–3975.72)	0.633	2229.06(1545.08–4063.76)	2810.99(1657.68–4302.99)	0.471
IL-1ra (pg/mL)	259.38(172.26–472.42)	235.02(173.44–338.66)	0.392	573.05(242.52–1221.935)	683.20(307.66–848.79)	0.982
IL-1β (pg/mL)	20.12 ± 14.85	17.70 ± 3.85	0.533	17.73 ± 5.30	17.38 ± 4.42	0.852
IL-6 (pg/mL)	3.28 ± 0.77	3.44 ± 1.53	0.697	4.51 ± 3.39	6.52 ± 3.49	0.534
IL-7 (pg/mL)	10.35 ± 3.29	13.56 ± 4.34	**0.045**	13.68 ± 7.17	16.98 ± 9.87	0.368
IL-15 (pg/mL)	6.76 (5.49–8.59)	6.98 (5.80–10.84)	0.626	7.86 ± 3.12	6.64 ± 2.19	0.634
Kallikrein 3 (pg/mL)	305.92 ± 82.54	329.22 ± 131.11	0.597	306.56 ± 102.06	357.96 ± 74.04	0.206
Kallikrein 5 (pg/mL)	1045.93 ± 476.68	835.53 ± 569.11	0.346	1099.09 ± 600.28	984.74 ± 735.35	0.714
Kallikrein 6/Neurosin (pg/mL)	1473.92(1273.09–1647.58)	1363.65(920.17–1742.88)	0.238	1504.69(1064.80–1826.89)	(1409.67(–798.61–1939.33)	0.451
Synuclein-alpha (pg/mL)	198.22(156.91–261.58)	209.01(139.67–327.54)	0.569	240.16(193.88–330.00)	268.66(209.57–326.97)	0.589

*ALB = Albumin; ALP = Alkaline phosphatase; ALT = Alanine aminotransferase; AST = Aspartate aminotransferase; CHOL = Cholesterol; CK = Creatine kinase; CREA = Creatinine; CysC = CystatinC; DBIL = Direct bilirubin; eGFR = Estimated glomerular filtration rate; GGT = Glutamyl transpeptidase; GLU = glucose; GLB = Globulin; HBDH = Hydroxybutyrate dehydrogenase; HDLC = High-density lipoprotein cholesterol; IBIL = Indirect bilirubin; K = Potassium; LDH = Lactate dehydrogenase; LDLC = Low-density lipoprotein cholesterol; NA = Sodium; TBIL = Total bilirubin; TG = Triglyceride; TP = Total protein; UREA = Urea; URIC = Uric acid. The bold values represent the P value less than 0.05.*

### Core Variable Selection and Establishment of the Identification Model

We investigated the possibility of identifying MSA-C patients based on candidate variables. Using the Lasso regression analysis for multivariate analysis, five core variables (AO, DBIL, AST, eGFR, and α-synuclein) were selected out of 47 possible indicators to formulate a disease panel. DBIL presented no significant differences between the two groups when assessed in univariate analysis. However, higher AST level (*P* = 0.027) and lower level of eGFR were observed in the MSA-C patients (*P* < 0.001). The remaining five core variables with favorable identification efficiency were integrated into a logistic identification model and simultaneously credited with weighting coefficients. Afterward, the five core variables were combined according to the weighting coefficients to obtain a scoring formula.

### The Performance of the Model

**FIGURE 2:**
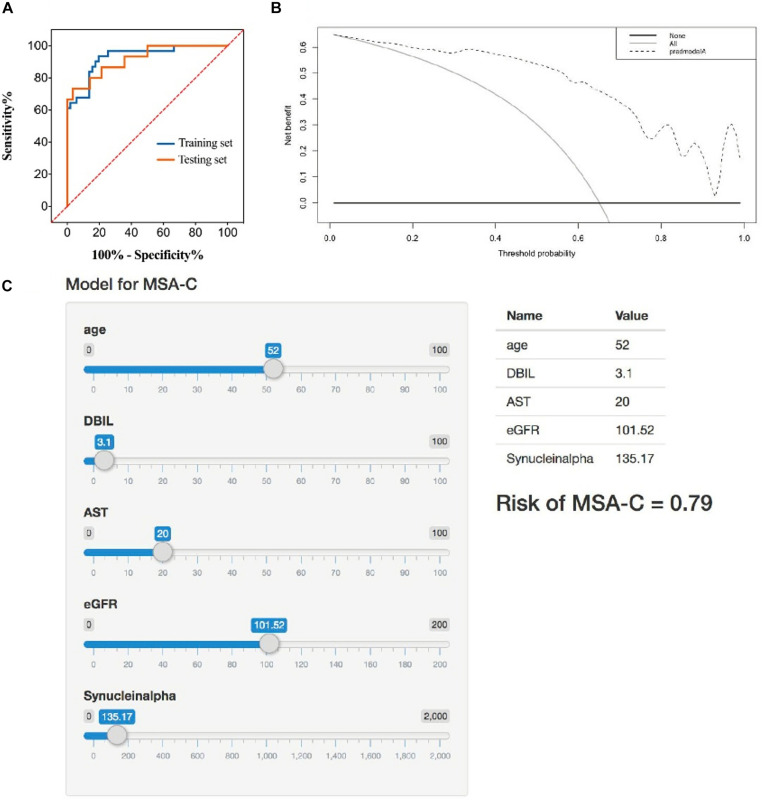
**(A)** Receiver operator characteristic curve of the identification model in training set and testing set. The AUC of this model is 0.929 and 0.917 in training set and testing set, respectively. **(B)** Decision analysis curve of the identification model. Dotted line: prediction model. Solid line: all patients with MSA-C. Horizontal line: all patients without MSA-C. The decision curve shows that using the identification model to identify MSA-C yields more benefits than total or no relative treatment. If the patient has a personal threshold probability of 60% (i.e., if the patient has a MSA-C probability of 60%, the patient will choose corresponding treatment), then the net benefit is 0.453 when the decision is made using the model. **(C)** Application example of the identification model. A 52-year-old male patient with suspected ataxia was admitted to the Department of Neurology, West China Hospital. We entered the corresponding parameters of each marker. Then, the model showed his probability of MSA-C was 0.79. The follow-up clinical comprehensive evaluation, neuroimaging examination, and genetic testing confirmed the speculation of our model.

The ROC curve was displayed to validate the predictive accuracy of the model. The ROC illustrated that an AUC of 0.929 (95% CI: 0.872–0.985) was present in the training set, and an AUC of 0.917 (95% CI: 0.829–0.995) was present for the testing set, revealing good concordance and reliable ability. The cutoff value for the training set was 0.707. The DCA quantitatively demonstrated a high clinical net benefit over the entire probability threshold (Figures 2A,B).

### External Independent Validation

We included 25 suspected ataxia patients to independently validate the model before obtaining their final definite diagnosis information. According to the suggestive prediction results from the model, 15 individuals were identified as MSA-C patients. Subsequently, we compared the model prediction results after obtaining the final diagnoses, which were confirmed using a combination of clinical evaluation, neuroimaging results, and genetic testing. The comparison revealed that 13 MSA-C patients were confirmed to have MSA-C compared with the predicted results of 15 individuals (13/15, positive predictive value = 86.67%). Two patients who were not recognized by the model were confirmed as MSA-C patients after the comprehensive diagnostic evaluation (8/10, negative predictive value = 80%).

### Construction of the Web Application

The Shiny R Package was used to transform the prediction model into a visualizing and operational web application^1^, which integrated all five selected factors. By dragging the slider below each of the variables, the corresponding parameter could change, and the sum of the points calculated represented the predictive probability of the risk for MSA-C (Figure 2C).

## Discussion

Over the past decade, many clinicians have summarized disease characteristics and conducted research with the goal of better defining and diagnosing MSA-C (Koga and Dickson, 2018). In fact, on account of heterogeneity in clinical characteristics due to different stages of disease and individual variation, it is easy to misdiagnose MSA-C as other similar diseases such as SCA. Meanwhile, with the lack of pedigree and genetic information, the certain diagnosis of SCA can also be difficult. However, little has been gained due to a lack of sufficient specific biomarkers of the disease. Unfortunately, no specific biomarkers for MSA-C have been found in this study or previous studies. Even though some potential specific biomarkers in our study exhibited significant differences, their specificity for a diagnosis of MSA-C was not convincing.

When specific biomarkers cannot meet the requirements for adequate disease diagnosis, a clinical prediction model based on information, including multiple demographic characteristics, clinical variables, and laboratory indicators, might improve the diagnostic efficiency for some neurological diseases, avoid specific biases, and provide relatively objective predictions. For example, a nomogram developed by Wei et al. based on seven predictive factors (the AO, rate of disease progression, hemoglobin A1c level, body mass index, creatinine, creatine kinase, and non-invasive positive pressure ventilation) was used to predict the possibility of longer survival of amyotrophic lateral sclerosis patients and attained an AUC of 0.92 (95% CI: 0.88–0.96) (Wei et al., 2018). Such advances also have been proposed and proved sufficient in the diagnosis and subsequent health care management of many diseases. Therefore, we hypothesized that combining variables from different assessment parameters could be used to develop successful predictive models to identify MSA-C patients.

In this study, we screened five predictors (AO, DBIL, AST, eGFR, and α-synuclein) as a panel that were combined to construct a predictive diagnosis model for MSA-C. These five predictors were essential for improving the identification of MSA-C patients. AO was an independent positive indicator for MSA-C, which matched the natural baseline information reported for MSA-C and SCA, as the peak AO of MSA-C was later than SCA (Jellinger and Wenning, 2016). Both AST and eGFR presented significant differences between the MSA-C and SCA groups, whereas there was no difference for DBIL between the two groups. Accumulative evidence has suggested misfolded α-synuclein could be a key component in the pathogenic pathway leading to neurodegeneration and the pathological presence in autopsy results of α-synuclein-containing protein aggregates, also known as glial cytoplasmic inclusion (GCI) bodies, was regarded as the crucial method for a definitive diagnosis of MSA (Trojanowski et al., 2007; Ubhi et al., 2011; Jellinger and Wenning, 2016; Woerman et al., 2018). Therefore, numerous studies have focused on CSF or blood α-synuclein levels in the diagnosis of MSA, but the results have been inconsistent. Interestingly, α-synuclein alone did not exhibit a significant difference between the two groups in our study. Nevertheless, it remained as one of the core variables suitable to be added into the model construction. The vast majority of MSA-C patients do not have a familial predisposition, and the family history of some patients were unclear or missing, so we did not include family history as a parameter in the variable-based prediction model.

The performance evaluation and external clinical validation for this model demonstrated good reliability and accuracy, with a satisfactory AUC of 0.929 and 0.917 in the training and testing sets, respectively. Only minor differences were observed between the two sets, all of which revealed the good discrimination accuracy of this model. Moreover, we performed a DCA evaluation in this study, and it indicated that the model had an overall high net clinical benefit at different threshold probabilities, suggesting that the judgments made in the model will benefit patients in most cases.

Also, determination of the true clinical application ability was of utmost importance, for which we enrolled 25 suspected ataxia patients as an independent validation cohort. The model results were compared with the comprehensive assessments for the 25 individuals, including family history, clinical manifestations, neuroimaging features, and genetic sequencing results. The model identification results demonstrated a relatively high predictive accuracy value, suggesting promising use in clinical practice. However, four patients were misclassified, among whom there was one ataxia patient with an undefined cause and one SCA patient.

It was notable that the five core variables, which may not present with statistic differences as single biomarkers in univariate analysis, were automatically chosen by the Lasso as a group with the best performance for differential identification. Lasso helped screen the potential predictors as well as maintain the objectivity, comprehensiveness, and accuracy, in view of balancing the number of variables and sample size at the same time. The inconsistency observed between the univariate analysis and multivariate analysis might result from the sample size, the number of variables, the interaction of multiple markers as a whole, or other factors. Therefore, in the future, we need to combine more elements and research as well as enroll more ataxia patients with other probable causes to improve the performance of the model.

At present, the prediction model cannot prove the causality between markers and the pathogenesis of the disease, but it theoretically and statistically displayed a certain correlation between markers and disease, which provide the clue for further fundamental researches. In our study, five core variables were integrated in a multi-parameter combination. Biologically speaking, bilirubin is related with oxidative stress. It plays a role in defending against the increased oxidative stress and some studies have suggested that low bilirubin levels and oxidative stress could occur in some neuroinflammatory diseases and neurodegenerative diseases (Ilzecka and Stelmasiak, 2003; Vitek, 2013). Previous study showed that TBIL and IBIL were lower in MSA patients than in healthy controls (Zhou et al., 2016). ALT and eGFR are indicators reflecting kidney and liver function respectively, and their roles in neurodegenerative diseases have also been reported (Nam et al., 2019; Nho et al., 2019; Palma et al., 2020). In fact, previous studies have suggested that chronic diseases such as diabetes mellitus (DM), hypertension, and depression may be associated with an increased risk of developing PD (Ascherio and Schwarzschild, 2016). However, none of the similar study for MSA has been found. Therefore, the metabolic and hormonal disturbances may be a topic of interest for further research of MSA-C.

Furthermore, based on the results mentioned previously, we translated the prediction model into a visual and operational web application, which can be applied to mobile devices. By dragging the slider to change the corresponding parameters, the point total is displayed automatically, which represents the probability of a diagnosis of MSA-C. The short time taken to detect the factors needed, the ease of use, and the capability for continuous optimization have made this application accessible and convenient for users.

However, this study presented several limitations. Because the study was restricted by the morbidity of MSA-C patients, the number of participants included in our study from a single center was small and might not accurately represent MSA-C patients as a whole. Even though we enrolled the MSA-C patients exclusively based on clinical diagnostic criteria without postmortem evidence, some bias could have been introduced when we chose the patients that were included in our study. The candidate biomarkers were limited. Additional biomarkers combined with neuroimaging features or other types of objective markers might provide a better process for the differential diagnosis of MSA-C. As for the SCA patients enrolled, due to the low prevalence of SCA, only the subtypes of SCA1, SCA2, SCA3, and SCA6 were included as a whole. Although SCA3 patients were in the majority of the controls, still the existence of heterogeneity might have a certain influence on the comparison of variables between two groups afterward on the efficiency and generalization of the model. The impact of the diversity of SCA subtypes can be further analyzed for the optimization of the model. Also, other types of ataxia-like sporadic adult-onset ataxia could be included as disease controls to improve the specificity of the model for MSA-C diagnosis. Therefore, we intend to add and analyze more variables from diverse aspects to accurately and efficiently differentiate MSA-C from other kinds of diseases to perfect this model. The model also needs to be validated using a larger population followed by a series of consistent development actions to expand the usability and reliability for application. After the dynamic detection of candidate biomarkers, this model also should be of considerable benefit to monitor and predict disease development.

## Conclusion

To our knowledge, this is the first study to establish a clinical prediction model based on demographic and laboratory variables selected by LASSO regression analysis, including AO, DBIL, AST, eGFR, and α-synuclein, for better differentiation between MSA-C and SCA, and the model presented excellent overall availability in our specific study group. It is highly anticipated that after continued improvement of the model and its validation in a larger population, it will be applied clinically as an integral auxiliary tool to assist in the differential diagnosis of MSA-C and advance related healthcare management.

## Data Availability Statement

The datasets analyzed in this article are anonymous to protect patient privacy and are not publicly available. Request to access the datasets should be directed to email the corresponding author.

## Ethics Statement

The protocol of this study was approved by the West China Hospital, Sichuan University Medical Ethics Committee. Written informed consent was obtained from all participants.

## Author Contributions

SG and MW designed the research and wrote the manuscript. BZ and YuZ responsible for the recruitment of patients with ataxia and neurological testing. YA and ZM responsible for the detection of candidate biomarkers. YaZ and MZ responsible for collecting and organizing data. DY responsible for neuroimaging assessment. BY supervised the experiment and revised the manuscript. All authors contributed to the article and approved the submitted version.

## Conflict of Interest

The authors declare that the research was conducted in the absence of any commercial or financial relationships that could be construed as a potential conflict of interest.

## Footnotes

1 https://guoshuo.shinyapps.io/shuo/

## Supplementary Material

Supplementary Figure 1 Concentration of twenty cytokines measured by Human Magnetic Luminex Assays in training set.

Supplementary Table 1 The methods for the 25 related metabolic tests in clinical laboratory.

Supplementary Table 2 The demographic and clinical information of SCA patients.

Supplementary Text The principles of Human Magnetic Luminex Screening Assay offered by the manufacturer (R&D Systems, Minneapolis, MN, United States).

## References

[B1] Ascherio A. Schwarzschild M. A. (2016). The epidemiology of Parkinson’s disease: risk factors and prevention. *Lancet Neurol.* 15 1257–1272. 10.1016/s1474-4422(16)30230-7 27751556

[B2] Chen D. Wei X. Zou J. Wang R. Liu X. Xu X. (2015). Contra-directional expression of serum homocysteine and uric acid as important biomarkers of multiple system atrophy severity: a cross-sectional study. *Front. Cell Neurosci.* 9:247. 10.3389/fncel.2015.00247 26217177 PMC4492156

[B3] Chitramuthu B. P. Bennett H. P. J. Bateman A. (2017). Progranulin: a new avenue towards the understanding and treatment of neurodegenerative disease. *Brain* 140 3081–3104. 10.1093/brain/awx198 29053785

[B4] Corey K. M. Kashyap S. Lorenzi E. Lagoo-Deenadayalan S. A. Heller K. Whalen K. (2018). Development and validation of machine learning models to identify high-risk surgical patients using automatically curated electronic health record data (Pythia): a retrospective, single-site study. *PLoS Med.* 15:e1002701. 10.1371/journal.pmed.1002701 30481172 PMC6258507

[B5] Dukic L. Simundic A. M. Martinic-Popovic I. Kackov S. Diamandis A. Begcevic I. (2016). The role of human kallikrein 6, clusterin and adiponectin as potential blood biomarkers of dementia. *Clin. Biochem.* 49 213–218. 10.1016/j.clinbiochem.2015.10.014 26515085

[B6] Fanciulli A. Stankovic I. Krismer F. Seppi K. Levin J. Wenning G. K. (2019). Multiple system atrophy. *Int. Rev. Neurobiol.* 149 137–192. 31779811 10.1016/bs.irn.2019.10.004

[B7] Gennarini G. Bizzoca A. Picocci S. Puzzo D. Corsi P. Furley A. J. W. (2017). The role of Gpi-anchored axonal glycoproteins in neural development and neurological disorders. *Mol. Cell Neurosci.* 81 49–63. 10.1016/j.mcn.2016.11.006 27871938

[B8] Gilman S. May S. J. Shults C. W. Tanner C. M. Kukull W. Lee V. M. (2005). The North American multiple system atrophy study, the north american multiple system atrophy study group. *J. Neural Transm. (Vienna)* 112 1687–1694. 10.1007/s00702-005-0381-6 16284910

[B9] Gilman S. Wenning G. K. Low P. A. Brooks D. J. Mathias C. J. Trojanowski J. Q. (2008). Second consensus statement on the diagnosis of multiple system atrophy. *Neurology* 71 670–676. 10.1212/01.wnl.0000324625.00404.15 18725592 PMC2676993

[B10] Greco I. Day N. Riddoch-Contreras J. Reed J. Soininen H. Kloszewska I. (2012). Alzheimer’s disease biomarker discovery using in silico literature mining and clinical validation. *J. Transl. Med.* 10:217. 10.1186/1479-5876-10-217 23113945 PMC3508881

[B11] Hsieh M. Hsieh B. Y. Ma C. Y. Li Y. T. Liu C. S. Lo C. M. (2019). Protective roles of carbonic anhydrase 8 in Machado-Joseph Disease. *J. Neurosci. Res.* 97 1278–1297. 10.1002/jnr.24474 31157458

[B12] Ilzecka J. Stelmasiak Z. (2003). Serum bilirubin concentration in patients with amyotrophic lateral sclerosis. *Clin. Neurol. Neurosurg.* 105 237–240. 10.1016/s0303-8467(03)00031-3 12954537

[B13] Jacobi H. du Montcel S. T. Bauer P. Giunti P. Cook A. Labrum R. (2015). Long-term disease progression in spinocerebellar ataxia types 1, 2, 3, and 6: a longitudinal cohort study. *Lancet Neurol.* 14 1101–1108. 10.1016/s1474-4422(15)00202-1 26377379

[B14] Jellinger K. A. (2017). Potential clinical utility of multiple system atrophy biomarkers. *Expert Rev. Neurother.* 17 1189–1208. 10.1080/14737175.2017.1392239 29023182

[B15] Jellinger K. A. Wenning G. K. (2016). Multiple system atrophy: pathogenic mechanisms and biomarkers. *J. Neural Transm. (Vienna)* 123 555–572. 10.1007/s00702-016-1545-2 27098666

[B16] Klockgether T. Ludtke R. Kramer B. Abele M. Burk K. Schols L. (1998). The natural history of degenerative ataxia: a retrospective study in 466 patients. *Brain* 121(Pt 4) 589–600. 10.1093/brain/121.4.589 9577387

[B17] Klockgether T. Mariotti C. Paulson H. L. (2019). Spinocerebellar ataxia. *Nat. Rev. Dis. Primers* 5:24. 10.1038/s41572-019-0074-3 30975995

[B18] Koga S. Dickson D. W. (2018). Recent advances in neuropathology, biomarkers and therapeutic approach of multiple system atrophy. *J. Neurol. Neurosurg. Psychiatry* 89 175–184. 10.1136/jnnp-2017-315813 28860330

[B19] Lee J. E. Song S. K. Sohn Y. H. Lee P. H. (2011). Uric acid as a potential disease modifier in patients with multiple system atrophy. *Mov. Disord.* 26 1533–1536. 10.1002/mds.23556 21542015

[B20] Mavroudis I. Petridis F. Chatzikonstantinou S. Kazis D. (2020). Alpha-synuclein levels in the differential diagnosis of lewy bodies dementia and other neurodegenerative disorders: a meta-analysis. *Alzheimer Dis. Assoc. Disord.* 34 220–224. 10.1097/wad.0000000000000381 32341240

[B21] Muzaimi M. B. Thomas J. Palmer-Smith S. Rosser L. Harper P. S. Wiles C. M. (2004). Population based study of late onset cerebellar ataxia in south east Wales. *J. Neurol. Neurosurg. Psychiatry* 75 1129–1134. 10.1136/jnnp.2003.014662 15258214 PMC1739172

[B22] Nam G. E. Kim N. H. Han K. Choi K. M. Chung H. S. Kim J. W. (2019). Chronic renal dysfunction, proteinuria, and risk of Parkinson’s disease in the elderly. *Mov. Disord.* 34 1184–1191. 10.1002/mds.27704 31021467

[B23] Nam G. E. Kim S. M. Han K. Kim N. H. Chung H. S. Kim J. W. (2018). Metabolic syndrome and risk of Parkinson disease: a nationwide cohort study. *PLoS Med.* 15:e1002640. 10.1371/journal.pmed.1002640 30130376 PMC6103502

[B24] Nho K. Kueider-Paisley A. Ahmad S. MahmoudianDehkordi S. Arnold M. Risacher S. L. (2019). Alzheimer’s Disease neuroimaging, and c. the Alzheimer Disease metabolomics, association of altered liver enzymes with Alzheimer Disease diagnosis, cognition, neuroimaging measures, and cerebrospinal fluid biomarkers. *JAMA Netw. Open* 2:e197978. 10.1001/jamanetworkopen.2019.7978 31365104 PMC6669786

[B25] Palma J. A. Norcliffe-Kaufmann L. Kaufmann H. (2018). Diagnosis of multiple system atrophy. *Auton. Neurosci.* 211 15–25. 29111419 10.1016/j.autneu.2017.10.007 PMC5869112

[B26] Palma J. A. Redel-Traub G. Porciuncula A. Samaniego-Toro D. Millar Vernetti P. Lui Y. W. (2020). The impact of supine hypertension on target organ damage and survival in patients with synucleinopathies and neurogenic orthostatic hypotension. *Parkinsonism Relat. Disord.* 75 97–104. 10.1016/j.parkreldis.2020.04.011 32516630 PMC7415666

[B27] Stefanits H. Wesseling C. Kovacs G. G. (2014). Loss of Calbindin immunoreactivity in the dentate gyrus distinguishes Alzheimer’s disease from other neurodegenerative dementias. *Neurosci. Lett.* 566 137–141. 10.1016/j.neulet.2014.02.026 24569123

[B28] Steiner J. Bogerts B. Schroeter M. L. Bernstein H. G. (2011). S100B protein in neurodegenerative disorders. *Clin. Chem. Lab. Med.* 49 409–424. 21303299 10.1515/CCLM.2011.083

[B29] Takae K. Hata J. Ohara T. Yoshida D. Shibata M. Mukai N. (2018). Albuminuria increases the risks for both Alzheimer Disease and vascular dementia in community-dwelling japanese elderly: the hisayama study. *J. Am. Heart Assoc.* 7:e006693. 10.1161/JAHA.117.006693 PMC5850144 29353232

[B30] Tibshirani R. (1997). The lasso method for variable selection in the Cox model. *Stat. Med.* 16 385–395. 10.1002/(sici)1097-0258(19970228)16:4<385::aid-sim380>3.0.co;2-3 9044528

[B31] Trojanowski J. Q. Revesz T. Neuropathology Working Group on Msa. (2007). Proposed neuropathological criteria for the post mortem diagnosis of multiple system atrophy. *Neuropathol. Appl. Neurobiol.* 33 615–620. 10.1111/j.1365-2990.2007.00907.x 17990994

[B32] Ubhi K. Low P. Masliah E. (2011). Multiple system atrophy: a clinical and neuropathological perspective. *Trends Neurosci.* 34 581–590. 10.1016/j.tins.2011.08.003 21962754 PMC3200496

[B33] van Waalwijk van Doorn L. J. Koel-Simmelink M. J. Haussmann U. Klafki H. Struyfs H. Linning P. (2016). Validation of soluble amyloid-beta precursor protein assays as diagnostic CSF biomarkers for neurodegenerative diseases. *J. Neurochem.* 137 112–121. 10.1111/jnc.13527 26748905

[B34] Vitek L. (2013). [Relationship of bilirubin to diseases caused by increased oxidative stress]. *Vnitr. Lek.* 59 618–621. 23909269

[B35] Watanabe H. Saito Y. Terao S. Ando T. Kachi T. Mukai E. (2002). Progression and prognosis in multiple system atrophy: an analysis of 230 Japanese patients. *Brain* 125 1070–1083. 11960896 10.1093/brain/awf117

[B36] Wei Q. Q. Chen Y. Chen X. Cao B. Ou R. Zhang L. (2018). Prognostic nomogram associated with longer survival in amyotrophic lateral sclerosis patients. *Aging Dis.* 9 965–975. 10.14336/ad.2017.1016 30574410 PMC6284758

[B37] Wenning G. K. Geser F. Krismer F. Seppi K. Duerr S. Boesch S. (2013). European multiple system atrophy study, the natural history of multiple system atrophy: a prospective european cohort study. *Lancet Neurol.* 12 264–274. 23391524 10.1016/S1474-4422(12)70327-7 PMC3581815

[B38] Woerman A. L. Watts J. C. Aoyagi A. Giles K. Middleton L. T. Prusiner S. B. (2018). alpha-Synuclein: multiple system atrophy prions. *Cold Spr. Harb. Perspect. Med.* 8:a024588. 10.1101/cshperspect.a024588 PMC5561534 28213437

[B39] Yabe I. Soma H. Takei A. Fujiki N. Yanagihara T. Sasaki H. (2006). MSA-C is the predominant clinical phenotype of MSA in Japan: analysis of 142 patients with probable MSA. *J. Neurol. Sci.* 249 115–121. 10.1016/j.jns.2006.05.064 16828805

[B40] Yamasaki R. Yamaguchi H. Matsushita T. Fujii T. Hiwatashi A. Kira J. I. (2017). Early strong intrathecal inflammation in cerebellar type multiple system atrophy by cerebrospinal fluid cytokine/chemokine profiles: a case control study. *J. Neuroinflammation* 14:89. 10.1186/s12974-017-0863-0 PMC5404297 28438224

[B41] Yokoyama T. Hasegawa K. Horiuchi E. Yagishita S. (2007). Multiple system atrophy (MSA) with massive macrophage infiltration in the ponto-cerebellar afferent system. *Neuropathology* 27 375–377. 10.1111/j.1440-1789.2007.00777.x 17899692

[B42] Zhou L. Jiang Y. Zhu C. Ma L. Huang Q. Chen X. (2016). Oxidative stress and environmental exposures are associated with multiple system atrophy in chinese patients. *Can. J. Neurol. Sci.* 43 703–709. 10.1017/cjn.2016.261 27670212

