# Supplementary figures and images for: Potential Fluid Biomarkers and a Prediction Model for Better Recognition Between Multiple System Atrophy-Cerebellar Type and Spinocerebellar Ataxia
Source: Front Aging Neurosci. 2021 Apr 20;13:644699. doi: 10.3389/fnagi.2021.644699 (PMC8093568; doi:10.3389/fnagi.2021.644699)

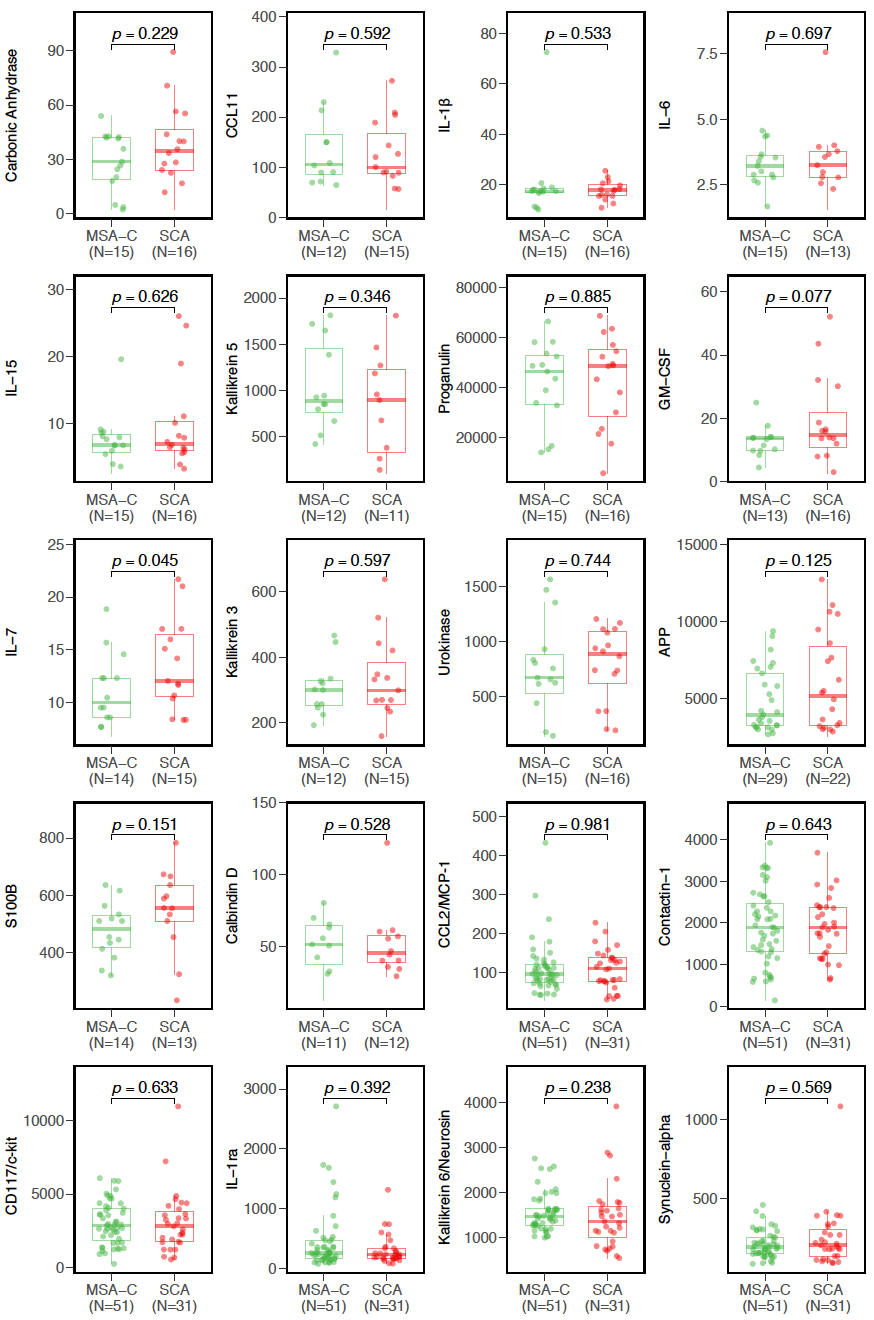

Supplement: Supplementary Figure 1 — Concentration of twenty cytokines measured by Human Magnetic Luminex Assays in training set. [file Image_1.JPEG]
