# Supplementary material for: Potential Fluid Biomarkers and a Prediction Model for Better Recognition Between Multiple System Atrophy-Cerebellar Type and Spinocerebellar Ataxia
Source: Front Aging Neurosci. 2021 Apr 20;13:644699. doi: 10.3389/fnagi.2021.644699 (PMC8093568; doi:10.3389/fnagi.2021.644699)
Supplement: Supplementary Table 1 — The methods for the 25 related metabolic tests in clinical laboratory. [file Table_1.docx]

**Table S1.** **The methods for t****he 25 related metabolic tests in clinical laboratory.**

| **Analytes** |  | **Methods** |
| --- | --- | --- |
| TBIL |  | UV spectrophotometry test |
| DBIL |  | UV spectrophotometry test |
| IBIL |  | UV spectrophotometry test |
| ALT |  | UV spectrophotometry test |
| AST |  | UV spectrophotometry test |
| TP |  | Colorimetric assay |
| ALB |  | Colorimetric assay |
| GLB |  | calculated by ( TP-ALB ) |
| GLU |  | UV spectrophotometry test |
| UREA |  | Kinetic test with urease and glutamate dehydrodenase |
| CREA |  | Enzymatic method |
| CysC |  | Immune turbidimetric test |
| URIC |  | Enzymatic colorimetric test |
| TG |  | Enzymatic colorimetric test |
| CHOL |  | Enzymatic colorimetric test |
| HDLC |  | Homogeneous enzymatic colorimetric test |
| LDLC |  | Homogeneous enzymatic colorimetric test |
| ALP |  | Colorimetric assay |
| GGT |  | Enzymatic colorimetric test |
| eGFR |  |  |
| NA |  | Ion Selective Electrode |
| K |  | Ion Selective Electrode |
| LDH |  | UV spectrophotometry test |
| HBDH |  | UV spectrophotometry test |
| CK |  | UV spectrophotometry test |

The 25 clinical related metabolic tests were performed by qualified laboratory personnels following standard operating protocols established by the Department of Laboratory Medicine in West China Hospital of Sichuan University **(WCH-LM-CHE-SOP-T1)**. And they were measured using Roche Cobas 702 automatic biochemical analyzer (Roche, Mannheim, Germany) with the corresponding reagents, calibrators, and quality control materials.
