# Supplementary material for: Potential Fluid Biomarkers and a Prediction Model for Better Recognition Between Multiple System Atrophy-Cerebellar Type and Spinocerebellar Ataxia
Source: Front Aging Neurosci. 2021 Apr 20;13:644699. doi: 10.3389/fnagi.2021.644699 (PMC8093568; doi:10.3389/fnagi.2021.644699)
Supplement: Supplementary Table 2 — The demographic and clinical information of SCA patients. [file Table_2.docx]

|  | **Training cohort** | | | | **Testing cohort** | | | | |
| --- | --- | --- | --- | --- | --- | --- | --- | --- | --- |
|  | SCA1 | SCA2 | SCA3 | SCA6 | | SCA1 | SCA2 | SCA3 | SCA6 |
| N | 3 | 3 | 24 | 1 | 2 | | 1 | 12 | 0 |
| Age of Onset | 36/43/54 | 29/47/52 | 43(36-54) | 52 | 28/49 | | 39 | 49(38-52) | / |
| Gender | 1/2 | 1/2 | 12/12 | 0/1 | 2/0 | | 1/0 | 6/6 | / |
| Family history | 2/2 | 3/3 | 21/24 | 0/1 | 1/2 | | 1/1 | 10/12 | / |
| Expanded CAG repeats | 47/51/52 | 37/37/39 | 67(63-70） | 23 | 51/55 | | 37 | 71(67-73) | / |
| Autonomic dysfunction | 0 | 0 | 15 | 0 | 0 | | 0 | 8 | / |
| Atrophy on MRI | 1 | 1 | 23 | 1 | 1 | | 1 | 9 | / |

**Table S2. The demographic and clinical information of SCA patients**

**Gender** was presented as *male/female*；**Family history** was shown as *cases with positive family history/ total cases with family history inquire；***Atrophy on MRI** refers to atrophy on MRI of putamen, middle cerebellar peduncle, pons, or cerebellum. Some data are presented as medians (upper and lower quartiles).
