# Supplementary material for: Potential Fluid Biomarkers and a Prediction Model for Better Recognition Between Multiple System Atrophy-Cerebellar Type and Spinocerebellar Ataxia
Source: Front Aging Neurosci. 2021 Apr 20;13:644699. doi: 10.3389/fnagi.2021.644699 (PMC8093568; doi:10.3389/fnagi.2021.644699)
Supplement: Supplementary Text — The principles of Human Magnetic Luminex Screening Assay offered by the manufacturer (R&D Systems, Minneapolis, MN, United States). [file Data_Sheet_1.docx]

**The principles of** **Human Magnetic Luminex Screening Assay offered by the** **manufacturer (****R&D Systems, Inc. Minneapolis, MN, USA)**

The kit of Magnetic Luminex^®^ Assays contains the components required to screen up the 20 proteins in serum samples in multiplexed sandwhich ELISAs. Analyte-specific antibodies are pre-coated onto magnetic microparticles embedded with fluorophores at set ratios for each unique microparticle regions. Microparticles, standards and samples are pipetted into wells and the immobilized antibodies bind the analytes of interest. After washing away any unbound substances, a biotinylated antibody cocktail specific to the analytes of interest is added to each well. Following a wash to remove any unbound biotinylated antibody, streptavidin-phycoerythrin conjugate Streptavidin -PE),which binds to the biotinylated antibodies, is added to each well. Final washes remove unbound Streptavidin-PE, the microparticles are resuspended in buffer and read using the Luminex MAGPIX Analyzer. A magnet in the analyzer captures and holds the superparamagnetic microparticles in a monolayer. Two spectrally distinct Light Emitting Diodes (LED) illuminate the microparticles. One LED excites the dyes inside each microparticle to identify the region and the second LED excites the PE to measure the amount of analyte bound to the microparticle. A sample from each well is imaged with a CCD camera with a set of filters to differentiate excitation levels.

The protocol was specifically provided for the Human Premixed Multi-Analyte kit with catalog number -LXSAHM.
